# Dementia service centres in Austria: A comprehensive support and early detection model for persons with dementia and their caregivers – theoretical foundations and model description

**DOI:** 10.1177/1471301213502214

**Published:** 2015-07

**Authors:** Stefanie R Auer, Edith Span, Barry Reisberg

**Affiliations:** M.A.S Alzheimerhilfe, Austria; Department for Clinical Neurosciences and Preventive Medicine, Danube University, Austria; M.A.S Alzheimerhilfe, Austria; Department of Psychiatry, New York University Langone Medical Center, USA

**Keywords:** caregiver support, persons with dementia, early disease detection, integrated care, nonpharmacological treatment of dementia

## Abstract

Despite the highly developed social services in Austria, the County of Upper Austria, one of the nine counties of Austria had only very limited specialized services for persons with dementia and their caregivers in 2001. Support groups existed in which the desire for more specialized services was voiced. In response to this situation, funding was received to develop a new structure for early disease detection and long term support for both the person with dementia and their caregivers. This article describes the development of the model of the Dementia Service Centres (DSCs) and the successes and difficulties encountered in the process of implementing the model in six different rural regions of Upper Austria. The DSC was described in the First Austrian Dementia Report as one of the potential service models for the future.

## Introduction

In recent years, pharmacological and nonpharmacological therapies (Olazaran et al., 2010) have emerged as strong reasons for structured professional approaches to persons with dementia (PwD). There is evidence that early diagnosis has multiple benefits both for the PwD and the support provider (SP) such as in preventing crises, facilitating disease adjustment, providing early access to a range of treatments and support (Prince, Bryce, & Ferri, 2011; Vernooij-Dassen, et al., 2005) and promoting appropriate and early care planning (Iliffe et al., 2002). The benefit of early disease detection and treatment on the prevention of premature institutionalization has been demonstrated (Koch & Iliffe, 2010; Mittelman, Ferris, Shulman, Steinberg, & Levin, 1996). Despite this encouraging evidence, only a fraction of PwD benefit from early disease detection and treatment (Banerjee et al., 2007; Bunn et al., 2012; World Health Organization, 2012). The National Dementia Strategy of England (Department of Health, 2009) identified three factors that might contribute to a low detection rate namely: (1) the stigma of dementia itself preventing open discussion and encouraging the withdrawal of affected persons and families; (2) the belief that memory problems are part of normal aging; (3) and the nihilistic perspective that nothing can be done. Early disease detection and the fight against stigma are the major goals of most National Alzheimer and Dementia Plans, for example the Australian National Framework for Action on Dementia (Alzheimer’s Disease International, 2012). Some national dementia strategies identify the general practitioner (GP) as well positioned to recognize the first symptoms of dementia. However, multiple barriers have been identified (Bunn et al., 2012; Koch & Iliffe, 2010; Vernooij-Dassen et al., 2005). Vernooij-Dassen et al. (2005) conclude that stigmatization by health care professionals including GP’s is the over-riding factor in delaying early diagnosis. Additionally, a reason for a delayed diagnosis in rural areas is a serious shortage of medical specialists (Turyn, 2001). The Scottish National Dementia Strategy (Alzheimer’s Disease International, 2012) emphasized that individuals were more likely to seek help if they believed supportive services would be readily available after diagnosis. Psychosocial support models for helping PwD and their SP dealing with the difficult post diagnostic disease phase have been developed. In the Netherlands, for example, the concept of “Meeting Centres” was developed (Dröes, Meiland, DeLange, Vernooij-Daassen, & Van Tilburg, 2003; Droes, Meiland, Schmitz, & Van Tilburg, 2004). In France as part of the French National Plan (2008), the concept of MAIA (Les Maisons pour l’autonomie et l’intégration des malades Alzheimer) was introduced. Both models, the meeting centres and the MAIA were developed as a one stop shop for SPs after the perception that services were offered in a fragmented fashion and therefore not reachable for burdened SPs and PwD (Droes et al., 2003; French National Plan, 2008). These centres follow the main goal of promoting social integration of both the PwD and the SP. One of the inclusion criteria for participating in a meeting centre is an already established medical dementia diagnosis (Dröes et al., 2003). In 2001, we started to develop a multicomponent psychosocial support model (Auer, Span, Donabauer, Reitner, & Helm, 2007) for PwD and their families living in rural areas, the Model of the Dementia Service Centre (DSC). In this model, early disease detection, diagnosis counselling, facilitating a medical diagnosis and the prevention of premature institutionalization are the main goals. In the first Austrian Dementia Report (Gleichweit & Rossa, 2009), the model of the DSC has been introduced as a possible future integrated support model for Austria.

### Austrian health and social care services

It is estimated that about 100 000 persons are currently affected by dementia in Austria and about one-third of affected persons receive a dementia diagnosis in the course of their illness (Gleichweit & Rossa, 2009). Recently, specialized services for PwD and their SPs are developing in Austria. Especially day care centres specializing in working with PwD from the middle stages onwards are being opened. Most of these day care centres however are offered in urban areas of the country. There is a seven step financial care scale providing financial support for families who assist or care for a relative with dementia at home. The scale has recently been adapted to better meet the needs of PwD in the beginning stages of the disease acknowledging assistive services (e.g. guiding somebody to perform a task). There are general social counselling and care counselling services providing guidance for nursing home placement. More and more care providers and mobile care services offer specialized dementia care and mobile social services (memory training, support for independent living). Nursing home care with increasing awareness of the special stage specific needs of PwD is offered but efforts are undertaken to keep persons at home as long as possible for example by providing mobile services. In Upper Austria, there are currently 188 medical specialists in neurology and psychiatry (Statistik Austria, 2013) available for about 1.4 million inhabitants.

### The development of the DSC Model

In 1997, a spouse caregiver founded the Morbus Alzheimer Syndrome (M.A.S) Association in the small rural town of Bad Ischl (Upper Austria). This initiative was privately financed and a support group for family members of PwD was established. At this time, there were neither specialized structures for social support for PwD and their SPs nor any specialized medical structures supporting an early medical dementia diagnosis in the county. In 2001, the association decided to take first steps to public funding and a clinical psychologist (*an* author of this paper) was hired for project development. The clinical psychologist started to work both with PwD and SPs, offering cognitive screening, counselling and an expert led caregiver support group. In the six month project preparation phase, the needs of both the PwD and their SPs were collected. The main issues voiced by PwD and their SPs were their insecurity with respect to the best time to receive a medical diagnosis and how to receive support even in the earlier stages of the disease. The caregivers also voiced a need to be informed about the disease and its implications and how to deal with difficult situations at home. The sorrow about their loved one’s decline without being able to do something was another main concern in the support groups. Some of the caregivers clearly indicated that placement in a nursing home was only an option as a last choice and that they intended to keep the PwD at home as long as possible. These needs made it very clear that the services provided needed to be specific to dementia and services developed for general social counselling were insufficient for the specific needs of PwD and their SPs. It also became clear that PwD and their SPs did not have the strengths to collect support services from different organizations by themselves and it was apparent that some were not even aware of the existence of such services. Taking all insights and current evidence into account, we developed the treatment model of the DSC with the central idea of a one stop shop for the PwD and their SP. With respect to the naming of the centres it was first considered that the term “Dementia” should be avoided since it was thought to foster stigmatization of PwD and their families. However, in order to clarify the specific target of the services provided, it was found necessary to employ, the word “Dementia”, which was therefore incorporated in the name of the structure. Seven methodological goals for the structure were defined (see Table 1).

**Table 1.** The defined goals of the DSC Model 1.

1.	Specifically designed for the needs of PwD and their SPs
2.	Easily accessed from the community
3.	Early disease detection through responding to the needs of the population
4.	One stop shop and information about other services available
5.	Long term individualized stage specific support and training for PwD and their families
6.	Development of a positive life concept
7.	Prevention of premature institutionalization

### Theoretical foundation of the treatment DSC Model

All elements of the treatment model are developed in a stage specific fashion. The stages used are described by Reisberg, Ferris, de Leon, and Crook (1982). The stage specific counselling methodology was stimulated by the concept of Mittelman, Epstein, and Pierzchala (2003). The theoretical fundamentals for the model can be best described as “humanistic” since it supports the potential of humans at all stages. The training methodology is theoretically framed by the theory of retrogenesis and its retrogenetic axioms (Reisberg et al., 2002; 2011). In these axioms, the fundamental capacities of humans, for example to learn, remain in a PwD throughout the entire disease process.

### Professionals working in the DSC Model

A DSC is run by a team consisting of a social worker (30 h per week) and a psychologist (20 h). Both professionals coordinate and supervise a group of about 10 trainers per DSC.

These trainers work either in the DSC or in the region where the person lives. In Table 2, the tasks of the professionals are listed.

**Table 2.** Professionals of a DSC and their tasks.

Social worker	Clinical psychologist	M.A.S trainer
Counselling (telephone or face to face)	Psychological screening and referral for medical diagnosis, Explaining medical reports to PwD and families	Creating an individualized training plan for a group/person
Coordination of appointments with families	Counselling (telephone or face to face)	Organizing/conducting group training or individual training (practical part)
Organizing/developing educational modules for SPs	Supervising trainers (content and quality of the training)	First person to be approached by SP in the training setting
Organizing/conducting support groups for SPs	Conducting SPs educational modules	Providing a training protocol for each training session
Organizing/coordinating training for PwD	Supervision of support groups
Organizing/coordinating available community services of other organisations

All professionals are specifically trained for their job in the DSC. A special curriculum lasting for one year was developed for the education of trainers (Schulz et al., 2012). Professionals hold regular team meetings and they receive regular professional supervision by a psychologist. There is a central DSC from which administrative tasks are performed (team management, data management, educational programs, funding issues, research activities). Regular regional and combined team meetings (all regional teams) are organised.

### Liaison with other professionals

The social worker’s role is to establish a network of referrals to all available additional services within the region of a DSC. His/her role is further to be helpful with formalities such as receiving or adjusting appropriate financial support through communications with community officials, or communicating with mobile services. It is the psychologist’s task to establish the network with the medical community of the region (hospitals, GP’s, medical specialists and competence centres for neurology and psychiatry). The psychologist communicates with the medical specialists personally and via a professional report providing the psychological test results. Attached to this report is the request for a medical diagnosis. Every effort is made to coordinate all available services for the families. All inter-professional communication needs to be authorized by the family.

### Public relations

In order to inform the community regarding the service, various public relation procedures have been instituted. Working against the stigma of dementia and the nihilistic perspective are the main messages. This is done with newspaper articles, lectures at all levels (community level up to university level lectures), TV and radio reports. The goal of all public relation activities is to provide a positive, hopeful image of the service (“something can be done”).

### The treatment pathway of a DSC

**Figure 1.**
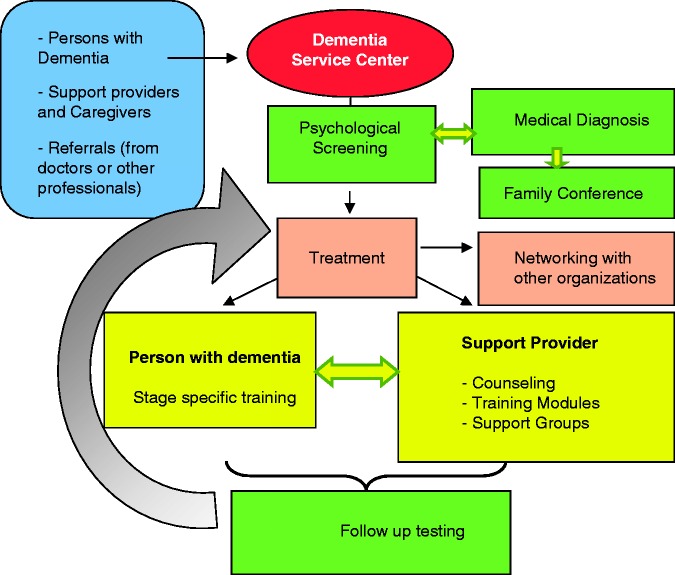
Illustration of the flow through the services of a Dementia Service Centre.

In Figure 1, the treatment pathway and service elements of the DSC model are depicted. Persons can approach the structure by themselves or they are informally referred to the DSC by physicians or other health care professionals.

### Welcoming persons into the model of a DSC

Most persons admitted to the DSC come in contact first via a telephone call. The social worker welcomes the person and explores the intention of the person calling. The purpose of the DSC is explained. If the inclusion criteria are met (i.e. person with memory problems), the person is invited for a personal contact. The social worker confines these interventions to the need of the person calling (for example if only information is required, this is all that is provided). If the person agrees, he/she is invited to a psychological screening (a “conversation” with the psychologist). If the person calling is the affected person, he/she is informed that they can bring a family member. The person may decline this request. The person is also asked to bring all medical records available to the first contact. If the person does not wish a psychological screening and wants to have information only, the social worker takes the telephone number and a regular telephone contact is agreed upon. In very uncommon instances, the person declines this option of a regular telephone contact as well.

### Psychological screening

For the psychological screening, a test battery consisting of the German Version of the Reisberg staging system (Ihl & Frölich, 1991) and the Mini Mental Status Examination (MMSE; Folstein, Folstein, & McHugh, 1975) is used. Further scales to assess the behavioural pathology in dementia, the BEHAVE-AD (Monteiro et al., 2001) and E-BEHAVE-AD (Auer, Monteiro, & Reisberg, 1996) are applied. Also included in the test battery are scales to assess caregiver depression (Sheikh & Yesavage, 1986) and caregiver burden (Bédard et al., 2001) as well as quality of life for both the PwD and the caregiver (Logsdon, Gibbons, McCurry, & Teri, 1999). For the testing procedure, a methodology was developed to use stage specific communication methods in the course of the testing in order to support the self-esteem of the PwD (Auer, Strotzka, Weber, Donabauer, & Psota, 2008). The test battery is administered at the person’s entry into the DSC (baseline) and every year after the baseline depending in part upon the participation of the person in the on-going stage specific training group or in the single session option. Persons without a medical diagnosis (Global Deterioration Scale (GDS) stages 3–7) are referred to a competence centre via a psychological report, to a medical practitioner or a private specialist in neurology or psychiatry. If the person is referred to a competence centre, the DSC receives a medical report in return. Reports are filed and if the person needs to return for medical follow-up or treatment, the PwD and the family are reminded to follow this suggestion. Any medical treatments, especially those related to the medical treatment of the symptoms of dementia, are monitored and the family is supported if they have questions or anxieties.

### The stage specific training for PwD

This training methodology (Auer, Gamsjäger, Donabauer, Span, 2010) was developed on the basis of the GDS staging system (Reisberg et al., 1993) and the theory of retrogenesis (Reisberg et al., 2002). Developmental age peer methodologies were adapted for each stage and tested for appropriateness. The training sessions are held using a “team approach”. Especially in the remote rural areas we employ the method of flexible group building. Wherever there is a cluster of at least three to four persons in an area, a local group opens, employing a trainer who also lives in this area using community rooms that are either available at no cost or can be rented at low cost. With this method, transportation costs can be saved. The acceptance level is high since local cultural aspects are respected (for example language intonation, habits and local customs). Therefore, the recruitment of trainers in the small regions is of great importance to save costs and to meet cultural needs.

### SP training and counselling

Training courses for SPs are organized on a regular basis. The topics of the training courses are the same for all centres and training materials are provided (e.g. power point presentations, literature lists for all professionals). The courses are usually held in the spring and the fall. The course consists of five modules held on five different occasions: (1) understanding dementia (course, symptoms); (2) principles of constructive communication; (3) doing activities at home; (4) social rights and financial support; (5) why respite for carers? Caregivers can bring the PwD to the modules and a trainer provides training during this time for the PwD. The second treatment element for SPs is the regularly provided support group (once a month). In addition, the social worker and the psychologist are available for personal counselling.

### The location of the centres

The centres are located in small towns or villages in the central area usually consisting of a common work room for staff, a separate room for psychological testing and a room for training groups big enough to perform physical training with about six persons. Training materials are kept in the DSC and loaned to the trainers. All elements of the treatment model including the organizational details and the team organization are described in a manual.

### Evaluation studies

After the developmental phase, a 3.5 year exploratory controlled study was conducted from 2002 to 2005. The control condition was care as usual with the option of counselling on request. The study was funded by the “Fonds Gesundes Österreich” (Project 599/III/83) and the County of Upper Austria and reviewed by the ethics committee of Upper Austria. A total of 141 PwD/SP dyads were recruited and 83 persons could be randomized to either the treatment or the control condition. The study showed a trend towards disease stabilization for the treatment group on the GDS (Reisberg et al., 1982) on the MMSE and on the functional assessment staging measure (FAST; Reisberg, 1988). There was no difference on the SP’s burden, as measured by the Zarit Burden Scale between the groups. On the basis of these results, two more centres were opened. Subsequently, the County of Upper Austria initiated two evaluation studies (Mechtler, 2008, 2010). These evaluation studies focused on the acceptance of the services and on the caregiver burden. Two hundred fifty questionnaires were sent out by mail to participating SP’s; 171 (69%) questionnaires were returned. Of the questionnaires which were returned, 66% reported a reduction of subjectively perceived care burden, 29% reported a persistent level of burden and 5% reported an increased burden in the time during their treatment in the DSC. The satisfaction with the services was high, 85% reported that they were satisfied with the service and 5% did not answer this question (Mechtler, 2008). The second evaluation study revealed similar positive results (Mechtler, 2010). In 2008, the M.A.S Alzheimerhilfe received a second grant, from the “Fonds Gesundes Österreich” (Project 1481/III/2) and the “County of Upper Austria” to study the implementation of three more Centres (Auer, 2010a). By 2009, six centres were operating throughout the county, exclusively in rural areas and positioned geographically to enable them to serve all rural county regions.

### The longitudinal database

A longitudinal database was initiated in 2002 consisting of 437 variables. The variable categories are listed in Table 3. Baseline and longitudinal data are collected for the PwD and for the SP. The data are organized within an Access Database. Data are entered by the teams of the six DSC via an internet based data management system.

**Table 3.** Variable categories in the data management system of the DSC.

Category	Numbers of variables
Basic sociodemographic data PwD	4
Contact data PwD/caregiver	19
Psychological test data	318
Illnesses	39
Medications	12
Social work data	21
Monitoring training sessions	8
Caregiver services	3
Caregiver training modules	2
Financial support	3
Time course	8
Sum	437

### Population characteristics

As of May 2013, 1796 persons (617 males and 1179 females) and their SPs received a baseline evaluation and agreed to be registered in the database. The median age of the population of PwD was 79 years (*Q*_1_72, *Q*_3_ 84). The median MMSE of the population was 21 (*Q*_1_16, *Q*_3_ 26). A total of 1766 GDS protocols from the baseline evaluation could be analysed. Fourteen persons were normal at their baseline (GDS stage 1), 209 persons had a subjective cognitive deficit (GDS stage 2), 264 persons had a mild cognitive impairment (GDS 3), 494 persons had a significant cognitive deficit (GDS stage 4), 538 persons had a moderate cognitive deficit (GDS stage 5), 225 had a severe cognitive deficit (GDS stage 6) and 22 persons had a very severe cognitive deficit (GDS stage 7). Of the 1796 recruited PwD, 1014 (56.5%) agreed to take part in the stage specific training and 782 (43.5%) did not participate. Families remained in the structure for a median of 1.9 years (median *Q*_1_ 0.9, *Q*_3_ 3.6), with extreme durations of over 11 years. As of May 2013, of the 1796 persons with a baseline, 659 persons reached an endpoint (death or institutionalization). Of the 659 persons with an endpoint, 298 persons were institutionalized (16.6% of the total population). About 80% of persons receiving a baseline evaluation also received a medical diagnosis of dementia in the course of their stay in the DSC. The DCS receives increasing numbers of direct referrals from hospitals and private practitioners. The advantage of monitoring medical issues and addressing the needs of the families is increasingly appreciated by the medical community.

### Funding and implementation of the model

The model of the DSC is still in the project phase and funded by the County of Upper Austria. All elements of the treatment model (screening for cognitive deficit, counselling, support group, teaching modules for caregivers) are free of charge because these services are delivered by the permanent team of the centres. A support fee of 10 Euro per hour for a training group session and 15 Euro for the single training session has to be paid by the PwD or the SP. The cost of one centre was calculated as 150 000 Euro per year. An implementation study is currently planned with the National Insurance Company and the County of Upper Austria. This study will start in the fall of 2013 and will end in December of 2014.

## Discussion

The success of the DSC in Upper Austria was made possible by the tremendous flexibility of the county administration in its willingness to try out new approaches. The novel aspect of this model is the element of the provision of a counselling and support phase before the dementia diagnosis. This service is offered to persons who approach the structure without a medical dementia diagnosis. A screening for the presence of a cognitive deficit is performed at the baseline evaluation in order to provide a specific referral for the medical specialist. This element was introduced because of the paucity of medical services available in the rural areas. In working with this population we realized that the acceptance of a dementia diagnosis is not an easy task for the individual. This diagnostic process often needs to be supported and the advantages for an early medical diagnosis need to be explained in order to be plausible for the PwD and the SP. The DSC model reaches a diagnosis rate of 80% of persons from GDS stage 3 (mild cognitive impairment) onwards. This appears to be very high in comparison with the diagnosis rate of the dementia population in the nation of Austria, where about 30% of affected persons are currently diagnosed. Considering the high percentage of persons in the early stages (GDS stages 3 and 4) of cognitive impairment presenting at the DSC and the overall percentage (80%) of a medical dementia diagnosis in the DSC, the structure is successful in both diagnosing dementia early and in obtaining a medical diagnosis. Our results support the observations presented in the literature (Bunn et al., 2012) with respect to the necessity of a pre-diagnostic support phase. The acceptance rate for the training is currently 56.5%. From the time of initiation of the DSC we noticed that about half of the population can be included in a stage specific training program. At this point it is not clear what factors influence the training participation and also, the attrition rate for the training program. There are factors of motivation both on the part of the PwD and the SP. Also, organizational factors influence the training participation such as the availability of groups in certain remote areas, transportation modalities and other factors. The factors hindering person’s participation in the training program require further study in order to both optimize entry and to minimize the training attrition rate. Our experience indicates that persons sometimes decide immediately after the initiation of the training to discontinue participation. However, most persons initiating training also remain in it for some time with the longest participation time being 11 years. These issues need further study. Droes, Meiland, Schmitz, and Van Tilburg (2004) report an institutionalization rate of 7.5% for the Meeting Centre Model in comparison with a 30.3% institutionalization rate for a day care support program after seven months of observation. Considering the substantially longer observational period of the DSC population, the 16% overall institutionalization rate for the DSC Model may be comparable to the institutionalization rate of the Meeting Centre Model.

Special care was taken in the development of stage specific services and methodologies for both the PwD and the SP in order to avoid negative consequences due to the observation of more severe disease symptoms in other persons. It is our experience that SPs and PwD pay special attention and can handle issues that arise in their current life situation. They are often disturbed by the observation of more severe disease symptoms and react by either withdrawing from the situation or becoming desperate and hopeless. Therefore, caregiver support groups and training groups are organized in a stage specific manner. The importance of stage specific service delivery (“the right service at the right time”) has been pointed out by other authors (Bruce & Paterson, 2000) and supports the individualized outlook on PwD in contrast to the delivery of services for “Dementia” in general. Acceptance of the model in the population of PwD and SPs was rapidly established. However, the acceptance within the helping community was not so easily achieved and required persistence. SP’s and PwD reported their frustration with traditional structures not being specifically interested in their needs. Therefore, we were motivated to close this service gap. Due to the extensive networking with professionals and the medical community surrounding the DSC, we observed an increase in awareness of medical specialists and GP’s with respect to the importance of early disease detection and dementia in general. The importance of this awareness in the medical community for early disease detection and the reduction of stigma has been noted in the literature (Vernooij-Dassen et al., 2005). Adding a medical doctor’s expertise in addition to a clinical psychologist may have an additional quality impact and shorten the time to receive a diagnosis. However, the cost of the service structure would substantially increase. Additional important issues have also became apparent. The team working in a DSC needs to be very well trained. Persons working in such a structure should be clear on a positive life concept and they should be able to “live” this attitude in their daily work. A well trained, enthusiastic and friendly (customer oriented) team is very important to transmit this positive life concept to PwD and their SP’s. Therefore, attention needs to be paid to the necessity of team meetings, supervision and the ongoing training of the team of service providers. We observed that families in need only call once. They take the initiative to call for help and if there is no response, they have the tendency to give up and call back in a crisis where help often comes too late and a nursing home admission cannot be prevented. Therefore, a properly structured welcome system is important.

Hurdles in serving rural areas have been reported in the literature (Beaulieu, Rowles, & Kuder, 2001; Turyn, 2001). Especially challenging was the set-up of training groups in very remote areas, where transportation can be very time-consuming and expensive. There are specific cultural needs in rural areas that prevent the usage of support services that have to be taken into consideration. Working very close to the community has helped to overcome some of the hardened community structures and attitudes with respect to the stigma of dementia making it possible for families to come forward without shame. We found that in addition to the strong and persistent public relations actions, word of mouth served the goals of the DSC. A business like “customer orientation” of dementia service programs is of great importance. Being a trainer was a good opportunity for employment in rural areas for women wishing to work for only a limited number of hours (for example women with small children, retired women). More and more men are also interested in working as trainers, for example, retired teachers. There are no volunteers currently working in the DSC Model. The culture of volunteering still needs to be nurtured in Austria and could help to lower costs of the structure in the future. Future efforts will focus on receiving funds for further analyzing the longitudinal database and for the detailed follow-up of persons not receiving stage specific training as a quasi-control group condition for the intervention in order to provide information on the effect of the DSC model on the prevention of premature institutionalization, and the course of dementia pathology.
